# Effect of metal catalyzed oxidation in recombinant viral protein assemblies

**DOI:** 10.1186/1475-2859-13-25

**Published:** 2014-02-17

**Authors:** Ricardo M Castro-Acosta, William A Rodríguez-Limas, Brenda Valderrama, Octavio T Ramírez, Laura A Palomares

**Affiliations:** 1 Departamento de Medicina Molecular y Bioprocesos, Instituto de Biotecnología, Universidad Nacional Autónoma de México, A.P. 510-3, C.P. 62210, Cuernavaca, Morelos, Mexico

**Keywords:** Protein oxidation, Carbonylation, Virus-like particles, Viral protein assemblies, Assembly efficiency

## Abstract

**Background:**

Protein assemblies, such as virus-like particles, have increasing importance as vaccines, delivery vehicles and nanomaterials. However, their use requires stable assemblies. An important cause of loss of stability in proteins is oxidation, which can occur during their production, purification and storage. Despite its importance, very few studies have investigated the effect of oxidation in protein assemblies and their structural units. In this work, we investigated the role of *in vitro* oxidation in the assembly and stability of rotavirus VP6, a polymorphic protein.

**Results:**

The susceptibility to oxidation of VP6 assembled into nanotubes (VP6_NT_) and unassembled VP6 (VP6_U_) was determined and compared to bovine serum albumin (BSA) as control. VP6 was more resistant to oxidation than BSA, as determined by measuring protein degradation and carbonyl content. It was found that assembly protected VP6 from *in vitro* metal-catalyzed oxidation. Oxidation provoked protein aggregation and VP6_NT_ fragmentation, as evidenced by dynamic light scattering and transmission electron microscopy. Oxidative damage of VP6 correlated with a decrease of its center of fluorescence spectral mass. The *in vitro* assembly efficiency of VP6_U_ into VP6_NT_ decreased as the oxidant concentration increased.

**Conclusions:**

Oxidation caused carbonylation, quenching, and destruction of aromatic amino acids and aggregation of VP6 in its assembled and unassembled forms. Such modifications affected protein functionality, including its ability to assemble. That assembly protected VP6 from oxidation shows that exposure of susceptible amino acids to the solvent increases their damage, and therefore the protein surface area that is exposed to the solvent is determinant of its susceptibility to oxidation. The inability of oxidized VP6 to assemble into nanotubes highlights the importance of avoiding this modification during the production of proteins that self-assemble. This is the first time that the role of oxidation in protein assembly is studied, evidencing that oxidation should be minimized during the production process if VP6 nanotubes are required.

## Background

Protein assemblies have gained increasing importance in the biomedical field, as they are used as vaccines, delivery vehicles and nanomaterials [1-4]. Viral proteins have a primary role in the field, as many of them are capable of self-assemble to form macromolecular structures with unique properties, such as virus-like particles (VLP) and other assemblies. The production of such complex structures can be challenging, as it is not sufficient to produce a pure protein, but the desired assemblies should be obtained in a reproducible and consistent manner [1,4]. Stability is a key property of assemblies that are to be used for pharmaceutical, biomedical or nanotechnological applications. However, proteins suffer modifications that can result in loss of stability and function. These modifications may be due to micro-environmental and environmental conditions and can occur during production, purification, formulation, storage and handling, causing irreversible changes in their quality and stability, such as deamidation, aggregation, mismatched S-S bonds and oxidation [5-7]. From these, oxidation is one of the most important, and therefore, most studied [8-10]. Oxidation has critical consequences for protein structure and function, disturbing intrinsic characteristics. *In vivo* protein oxidation has been related to several diseases, such as Alzheimer’s, cancer, atherosclerosis and other chronic disorders [10,11].

Protein oxidation has been extensively investigated by *in vitro* studies. Several groups have worked with proteins like bovine serum albumin (BSA), IgG, lysozyme, and human α1-antitrypsin, among others [12-16]. Oxidation reactions with 2,2′-azobis(2-amidinopropane) dihydrochloride (AAPH), H_2_O_2_, ^●^OH or O_2_^●-^ result in aggregation, structural damage, changes in physicochemical properties, cleavage, and changes in protein hydrophobicity and conformation [12-16]. Oxidative damage in proteins disturbs their three-dimensional structure due to accumulation of amino acid carbonylation, backbone fragmentation, cross-linking, unfolding, increase in hydrophobicity, and conformational modifications [8-10,17,18]. Only few reports have been published for viral protein macrostructure assemblies such as virus, virus-like particles, and other highly ordered assemblies. The effect of oxidation on viruses (adenovirus, bacteriophage MS2, cowpea mosaic virus, influenza virus and norovirus) has been studied in efforts to inactivate them [19-23]. Various sources of reactive oxygen species (ROS) have been tested, such as the Fenton reaction, UV_254_, ^1^O_2_, chlorine dioxide and free chlorine. ROS reacted with different sites on viral capsids, resulting in carbonyl formation, aggregation and conformational changes, as well as modifying the capsid cell binding capacity and diminishing virus infectivity [19-23]. However, to our knowledge, only a single report of the effect of oxidation on VLP exists. Tleugabulova et al. [24] studied the effect of oxidation on VLP of the hepatitis B surface antigen (HBsAg) oxidized with ammonium peroxodisulphate. Oxidation provoked VLP aggregation and cross-linking of S protein chains, leading to a complete loss of antigenicity. Such studies highlight the importance of further investigating the effect of aggregation on protein assemblies.

The structure of protein assemblies can result in an increased susceptibility to oxidation, as protein monomers are in close contact, which can result in chain reactions that could magnify the effect of ROS. Also, oxidation can damage protein assemblies, impede the assembly of monomers, or cause other alterations of the multimer. In this work, we investigated the effect of oxidation in a multimeric polymorphic protein, rotavirus VP6. Recombinant VP6 forms highly stable trimers that can self-assemble into different types of structures depending on pH and ionic strength [25,26]. VP6 assembled into trimers, nanotubes (VP6_NT_) or icosahedra can be obtained [25,26]. VP6 nanotubes have proven to be useful as a recombinant vaccine against rotavirus [2,27], as adjuvants in other recombinant vaccines [27], and as scaffolds for the production of nanomaterials [3,28]. VP6 constitutes an ideal model for studying the effect of oxidation on protein assemblies, as its assembly is required for its application. In this work, metal-catalyzed oxidation (MCO) was exerted upon VP6 nanotubes and unassembled VP6, in order to investigate its effect on protein degradation, carbonylation, assembly capacity, and aggregation. In this article, the susceptibility of oxidation of assembled and unassembled protein forms were compared, and the effect of oxidation on viral protein assembly is reported for the first time.

## Results

### VP6 nanotube characterization

**Figure 1:**
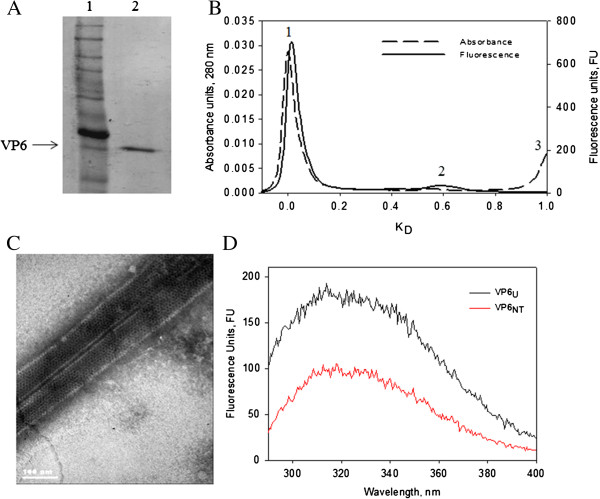
**Characterization of purified VP6**_**NT**_**. A)** 12% SDS-PAGE gel stained with Coomassie Blue (under reducing conditions): Lane 1, Molecular weight marker Benchmark (Life Technologies Corp., Carlsbad, CA, USA), the more intense band corresponds to 50 KDa. Lane 2, VP6_NT_. **B)** Size exclusion chromatography, Peak 1 corresponds to VP6_NT_, peak 2 to VP6_U_ and peak 3 to salts. K_D_ refers to the relative elution volume calculated with Equation 1. **C)** Transmission electronic micrograph at 85,000X. **D)** Intrinsic fluorescence spectrum of 40 µg/mL of VP6_NT_ or VP6_U_. Excitation at 280 nm.

A typical VP6 nanotube preparation was characterized. The purity of VP6 was confirmed in reducing denaturing SDS-PAGE gels, which showed a single band with the molecular weight reported for VP6 (Figure 1A). Size exclusion chromatography (SEC) analysis showed two populations (Figure 1B), one that migrated at the column exclusion limit (peak 1), which corresponded to VP6 nanotubes (VP6_NT_), and a second one with smaller size identified as VP6_U_ (peak 2), containing unassembled VP6 monomers and trimers. The population containing VP6_NT_ constituted 95% of the total protein, which is a typical value obtained with this purification process [3,29]. The presence of VP6_NT_ structures was confirmed by TEM (Figure 1C). Intrinsic fluorescence spectra of aromatic amino acids were acquired for VP6_NT_ and VP6_U_ at 280 (Trp and Tyr, Figure 1D) and 295 nm (Trp). VP6_U_ had a fluorescence quantum yield 2 and 2.5 times higher than VP6_NT_, at 280 and 295 nm, respectively.

### VP6 assembled into nanotubes is more resistant to degradation by oxidation than unassembled VP6 and BSA

**Figure 2:**
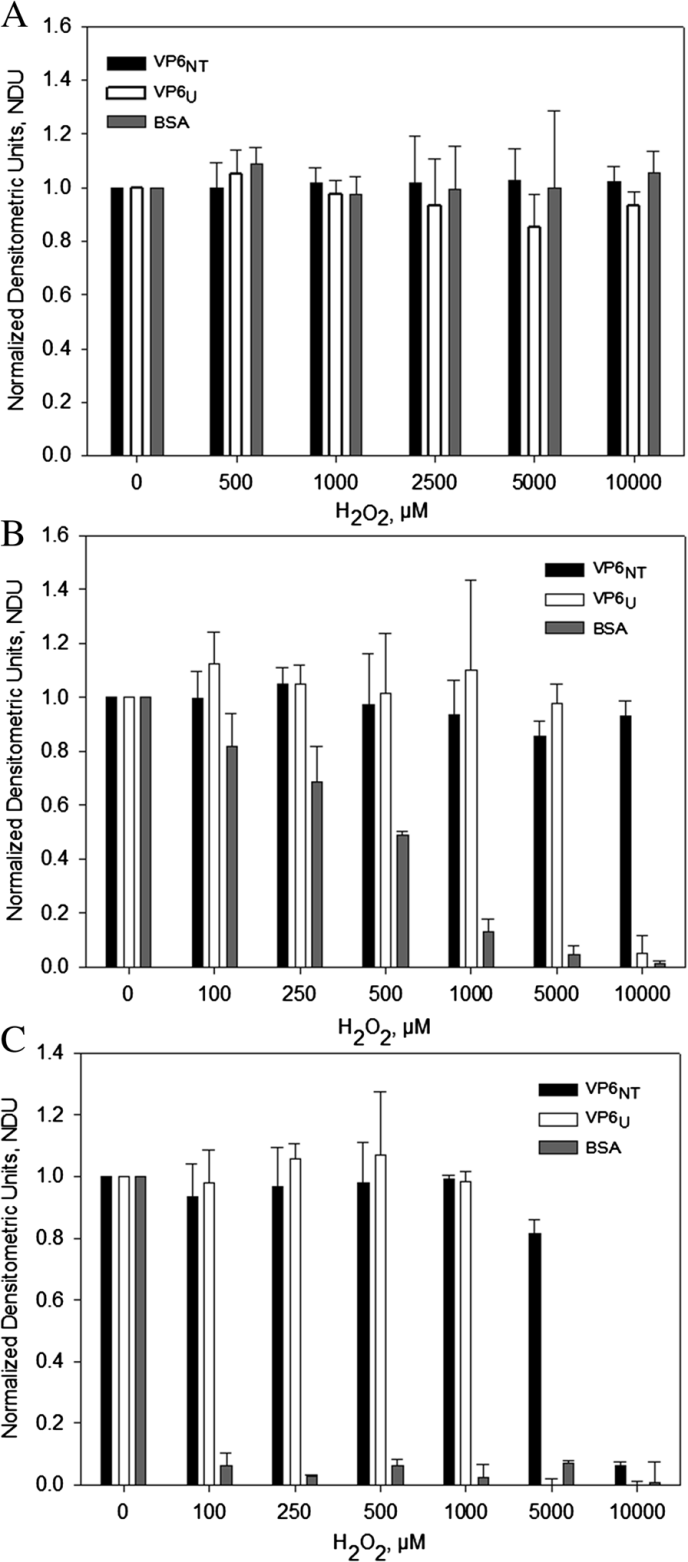
**Densitometric analysis of reducing 12% SDS-PAGE gels loaded with 2.5 µg of VP6**_**NT**_**, VP6**_**U **_**and BSA previously exposed to different oxidative treatments and stained with Coomassie blue. A)** Exposure of protein samples to different concentrations of H_2_O_2_ for 6 h. **B)** Exposure of protein samples to metal catalyzed oxidation (MCO) with 150 µM of FeCl_2_ at various H_2_O_2_ concentrations for 1 h. **C)** Exposure of protein samples to MCO with 150 µM of FeCl_2_ at various H_2_O_2_ concentrations for 6 h. Measurements were performed to identically treated samples from triplicate experiments. Error bars represent standard deviations among experiments.

Protein oxidation can result in degradation by fragmentation of the backbone, which can be evidenced by the disappearance of a stainable band in SDS-PAGE gels [15,17]. Degradation analysis was used to evaluate the susceptibility of nanotubes and disassembled VP6 to H_2_O_2_. For comparison, bovine serum albumin (BSA), a widely studied protein, was also subjected to oxidation. Gels were scanned and the intensity and area of each band were quantified by densitometry. Results are shown in Figure 2. Exposure to up to 10,000 µM H_2_O_2_ did not cause band disappearance in gels of treated BSA, VP6_NT_ or VP6_U_, even after 6 h of incubation with the oxidant (Figure 2A). As VP6 was not degraded by exposition to H_2_O_2_, all following experiments were performed only with MCO. In contrast, when exposed to H_2_O_2_ in MCO, the VP6 and BSA bands disappeared although with different behavior (Figures 2B and C). While VP6, in either of its forms, resisted MCO up to 5 mM of H_2_O_2_ for 1 h, the BSA band decreased at H_2_O_2_ concentrations above 0.25 mM. Exposition to H_2_O_2_ in MCO for 6 h caused degradation of BSA at all concentrations tested, evidencing that it is less resistant to degradation than VP6. These experiments also showed that VP6_NT_ are more resistant to oxidation than VP6_U_. While the VP6_U_ band disappeared after exposure to 10,000 µM of H_2_O_2_ in MCO for 1 h, no change was observed in VP6_NT_ when incubated under the same condition. Exposure of VP6_NT_ to high H_2_O_2_ concentrations for as much as six hours was needed for its band to disappear, suggesting a higher stability towards oxidative insults. The same behavior was observed in native gels (data not shown).

**Figure 3:**
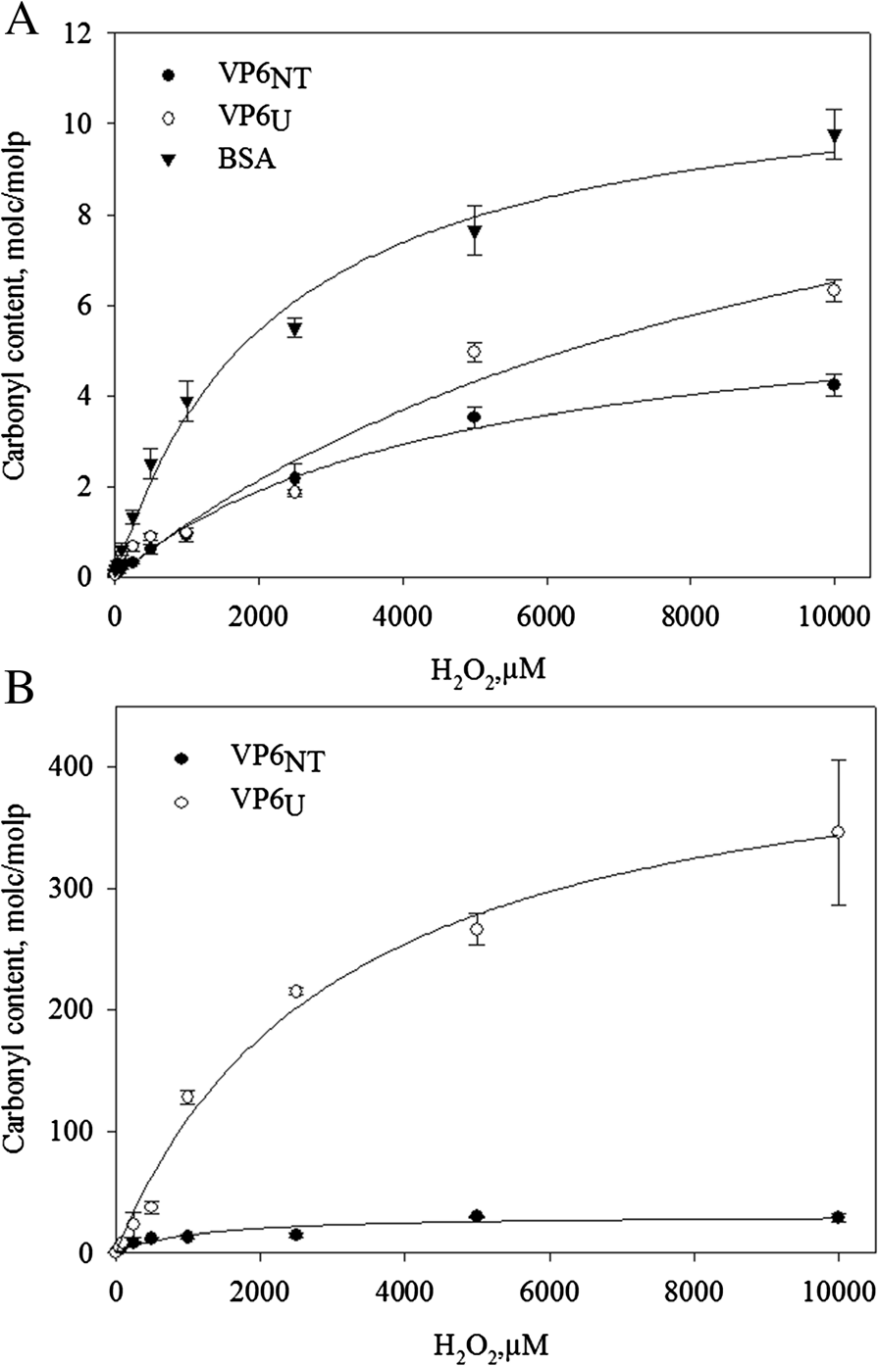
**Carbonyl content on VP6**_**NT **_**and VP6**_**U **_**after MCO. A)** MCO was performed with 150 µM of FeCl_2_ and different H_2_O_2_ concentrations for 1 h. Experiments were performed in triplicate and error bars represent the standard deviation between them. **B)** MCO with equimolar concentrations of FeCl_2_ and H_2_O_2_. Experiments were performed in duplicate, error bars represent the difference between them. Lines show the behavior described by equation (1) using the parameters listed in Table 1 for each condition.

In order to further dissect the molecular impact of the oxidative damage inflicted into VP6, the carbonyl content in VP6_U_, VP6_NT_, and BSA was measured after exposure to MCO at various H_2_O_2_ concentrations (Figure 3A). The initial carbonyl contents before oxidation were 0.069 ± 0.023 molc/molp for VP6_NT,_ 0.059 ± 0.023 molc/molp for VP6_U_ and 0.167 ± 0.010 molc/molp for BSA. Carbonyl content increased in all samples as H_2_O_2_ concentration increased, following a saturation curve. Results shown in Figure 3A were obtained maintaining the Fe^+2^ concentration constant at 150 µM while increasing H_2_O_2_ concentration to excess. This condition can result in the formation of oxidative species other than •OH, such as the remaining H_2_O_2_ or •OOH [8,12]. In order to restrict the formation of ROS to the •OH radical, the Fenton reaction was performed at equimolar concentrations of Fe^+2^ and H_2_O_2_ (Figure 3B). The carbonyl content under this condition was 64 times higher for VP6_U_ and 5 times higher for VP6_NT_ than when Fe^+2^ concentration remained constant. It also followed a saturation curve.

Data sets were adjusted to the following equation describing a saturation curve:

(1) $$\left[c\right]=\frac{{\left[c\right]}_{\text{max}}\left[H_{2}O_{2}\right]}{a+\left[H_{2}O_{2}\right]}$$

where [*c*] is the carbonyl content (molc/molp), [*c*]_*max*_ is the maximum carbonyl content, and *a* is a saturation constant. The values of the equation constants for each condition are listed in Table 1. The specific carbonyl content at saturation with constant Fe^+2^ was two times higher in VP6_U_ than in VP6_NT_, whereas [*c*]_*max*_ was similar for BSA and VP6_U_. In an analogy with enzyme kinetics, protein susceptibility to oxidation (affinity towards the oxidant) can be inferred from *a*. BSA was the most susceptible to oxidation, while the susceptibility of VP6_NT_ to oxidation was two times higher than the susceptibility of VP6_U_. At equimolar Fe^+2^ and H_2_O_2_ concentrations, a similar behavior was observed, where [*c*]_*max*_ and *a* were 14 and 2.6 times higher in VP6_U_ than in VP6_NT_, respectively.

**Table 1:** **Coefficients in Equation 1 obtained by fitting carbonyl content at different H**_**2**_**O**_**2 **_**concentrations**

**Sample**	**[c]**_**max**_**, molc/molp**	**a, µM**	**r**^**2**^
*At constant [Fe*^*+2*^*] and various [H*_*2*_*O*_*2*_*] (Figure*3*A)*			
VP6_NT_	6.40 ± 0.43	4,745.99 ± 681.64	0.99
VP6_U_	13.28 ± 3.72	10,431.12 ± 4824.19	0.97
BSA	11.47 ± 0.61	2,216.32 ± 324.21	0.99
*With equimolar H*_*2*_*O*_*2*_*/Fe*^*+2*^*concentrations (Figure*3*B)*			
VP6_NT_	31.49 ± 4.16	1,154.85 ± 499.22	0.89
VP6_U_	448.73 ± 32.19	3,064.28 ± 546.32	0.99

Data were fitted using the software SigmaPlot 10.0 (Systat Software, Inc., SJ, CA, USA). Coefficient standard errors are shown.

### Oxidation produced aggregation of VP6_U_ and fragmentation of VP6_NT_

**Figure 4:**
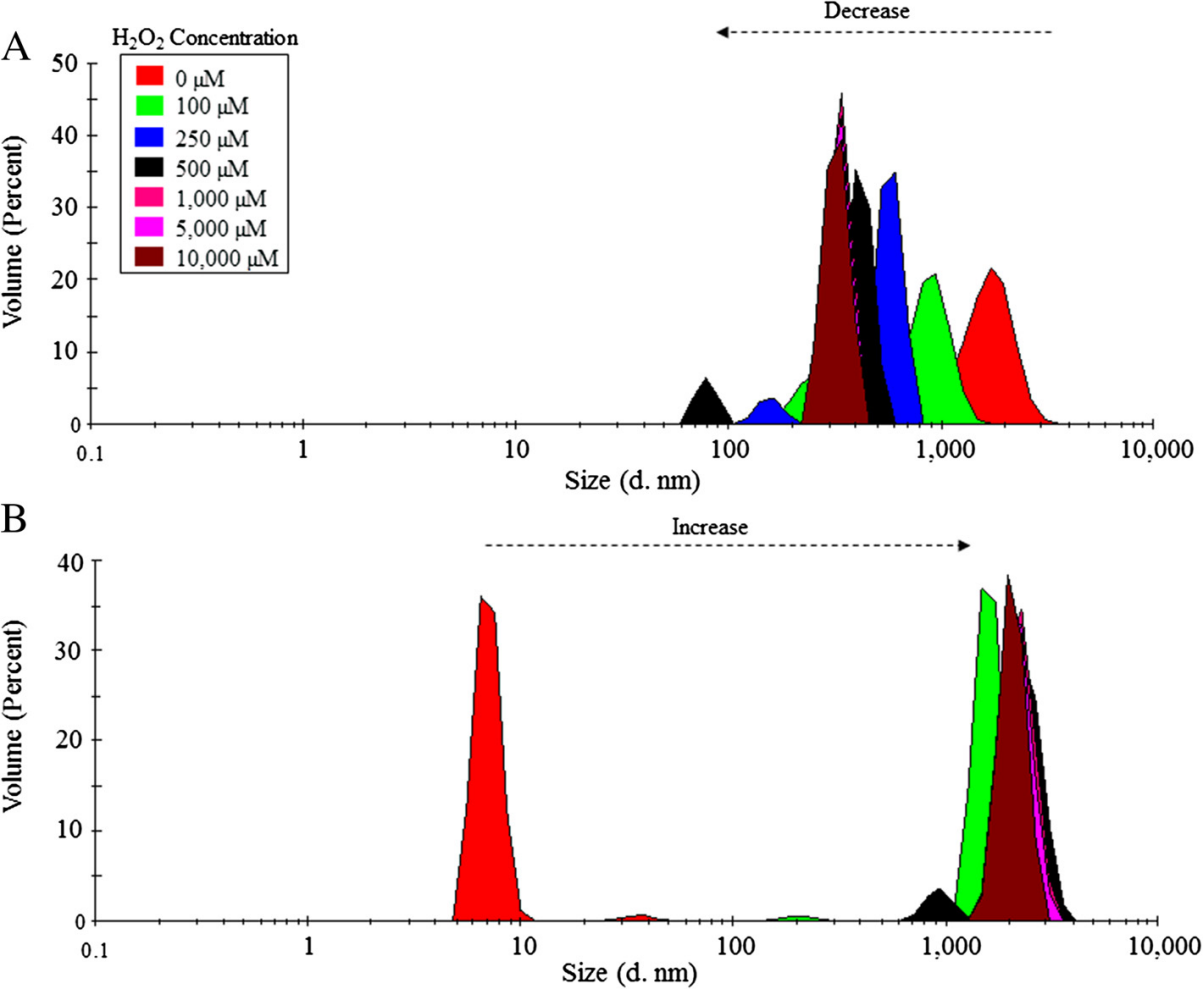
**Dynamic Light scattering (DLS) analysis of 0.4 mg/mL of VP6**_**NT **_**(A) and VP6**_**U **_**(B) exposed to MCO performed with 150 µM of FeCl**_**2 **_**and different H**_**2**_**O**_**2 **_**concentrations for 1 h.** Experiments were performed in triplicate, and a representative size distribution for each condition is shown.

**Figure 5:**
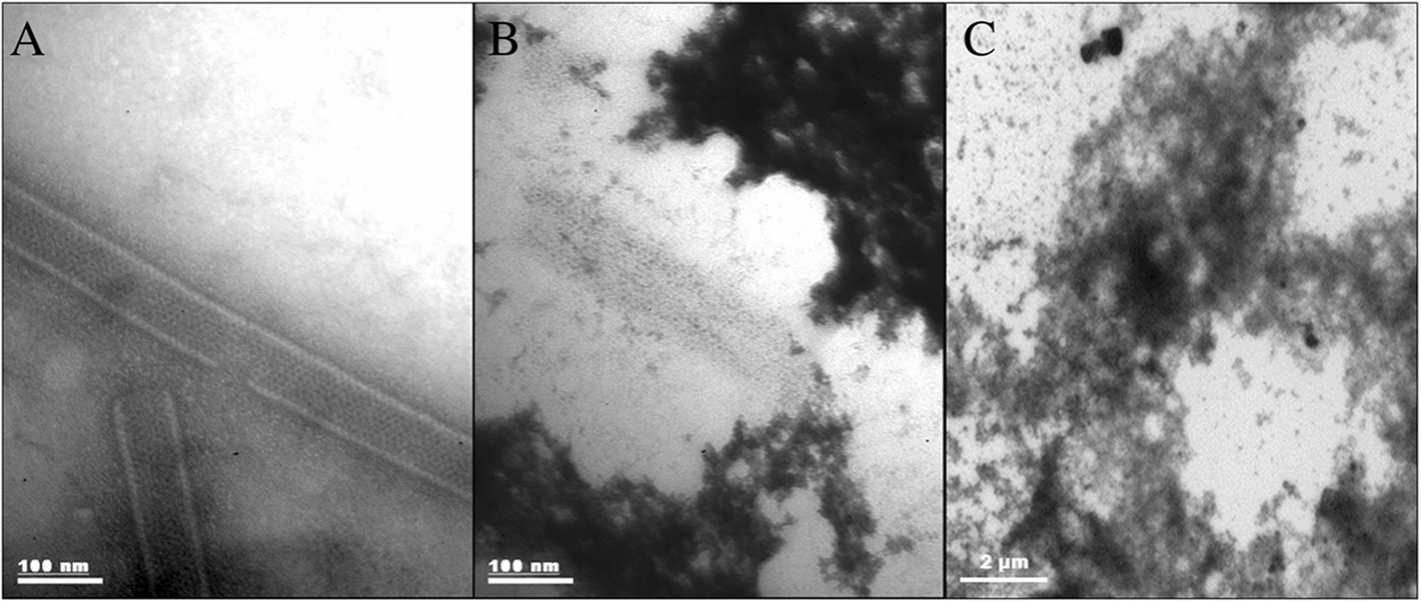
**Transmission electronic microscopy (TEM) of VP6 (0.4 mg/mL) after MCO at 10,000 µM of H**_**2**_**O**_**2 **_**and 150 µM of FeCl**_**2 **_**for 1 h.** Samples were stained with 2% uranyl acetate. **A)** Untreated VP6_NT_. **B)** Oxidized VP6_NT_ sample. **C)** Oxidized VP6_U_ sample.

Oxidized samples of VP6_NT_ and VP6_U_ were analyzed by dynamic light scattering (DLS), transmission electron microscopy (TEM), SEC and spectrometry. The size of VP6_NT_, measured by DLS, decreased as peroxide concentration increased (Figure 4A). The mean hydrodynamic diameter of nanotubes, which was 1,067.0 ± 206.6 nm (corresponding to an equivalent sphere) without oxidation, decreased down to 317.5 ± 40.4 nm after exposition to 10,000 µM H_2_O_2_. TEM showed that oxidation provoked the disassembly of nanotubes and aggregation of the resultant unassembled VP6 (Figure 5B). In contrast, oxidation of VP6_U_ resulted in an increase in size, from a hydrodynamic diameter of 7.5 ± 2.6 nm to 2,085.0 ± 289.7 nm after exposition to 10,000 µM of H_2_O_2_ (Figure 4B). TEM showed that the increase in size of oxidized VP6_U_ was caused by aggregation (Figure 5C).

Another consequence of protein oxidation is dityrosine formation [30], which could have caused the aggregation observed in both types of VP6 assemblies. No dityrosines were detected in either VP6_NT_ or VP6_U_ (data not shown), indicating that aggregation was caused by other mechanisms, such as hydrophobic interactions or hydrogen bonding [31,32].

**Figure 6:**
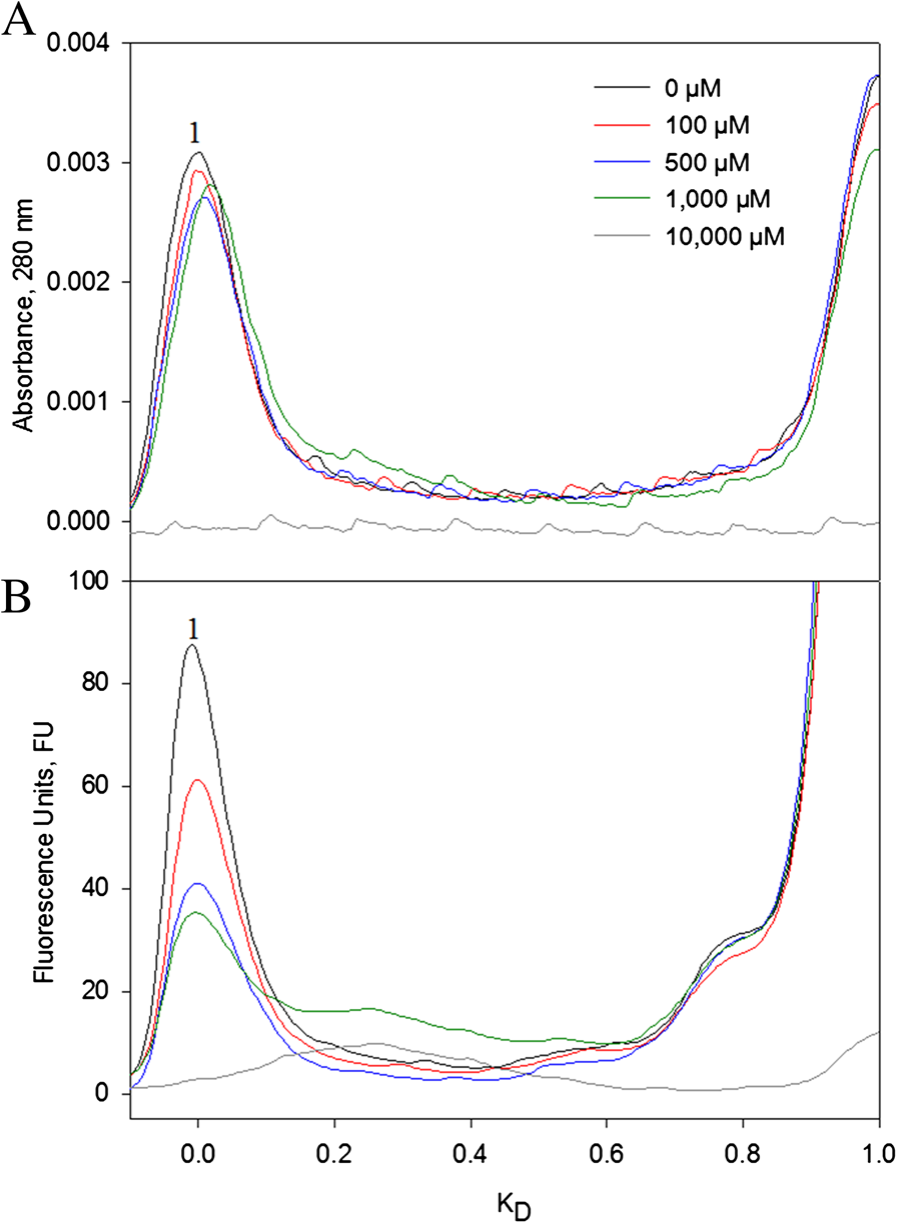
**Size exclusion chromatography of VP6**_**NT **_**samples (0.4 mg/mL) after MCO with 150 µM of FeCl**_**2 **_**at various H**_**2**_**O**_**2 **_**concentrations for 1 h. A)** Absorbance elution profile at 280 nm. **B)** Fluorescence elution profile at λ_ex_ 280 nm and λ_em_ 350 nm. Peak 1 corresponds to VP6_NT_. Experiments were performed in duplicate and a representative size distribution for each condition is shown.

To further understand the effect of oxidation in VP6_NT_, oxidized samples were analyzed by SEC, as previously described [29,33]. Absorbance at 280 nm and fluorescence of aromatic amino acids were followed (Figure 6). No changes in absorbance at 280 nm were observed in chromatograms when VP6_NT_ were oxidized with up to 1 mM of H_2_O_2_ (Figure 6A). Such a result was expected, as DLS analysis showed that the products of oxidation, although smaller than nanotubes, were larger than the column pore size (50 nm). However, no absorbance was detected after oxidation with 10 mM H_2_O_2_. In contrast with absorbance, fluorescence of aromatic amino acids decreased as H_2_O_2_ concentration increased. Fluorescence decreased 40% upon oxidation with 100 µM of H_2_O_2_, and consistently decreased until it completely disappeared at 10 mM (Figure 6B). Additional peaks were observed at 1 mM of H_2_O_2_, possibly smaller degradation products not detectable by absorbance.

### Oxidation provoked changes in fluorescence intensity and in the center of fluorescence spectral mass (CSM)

**Figure 7:**
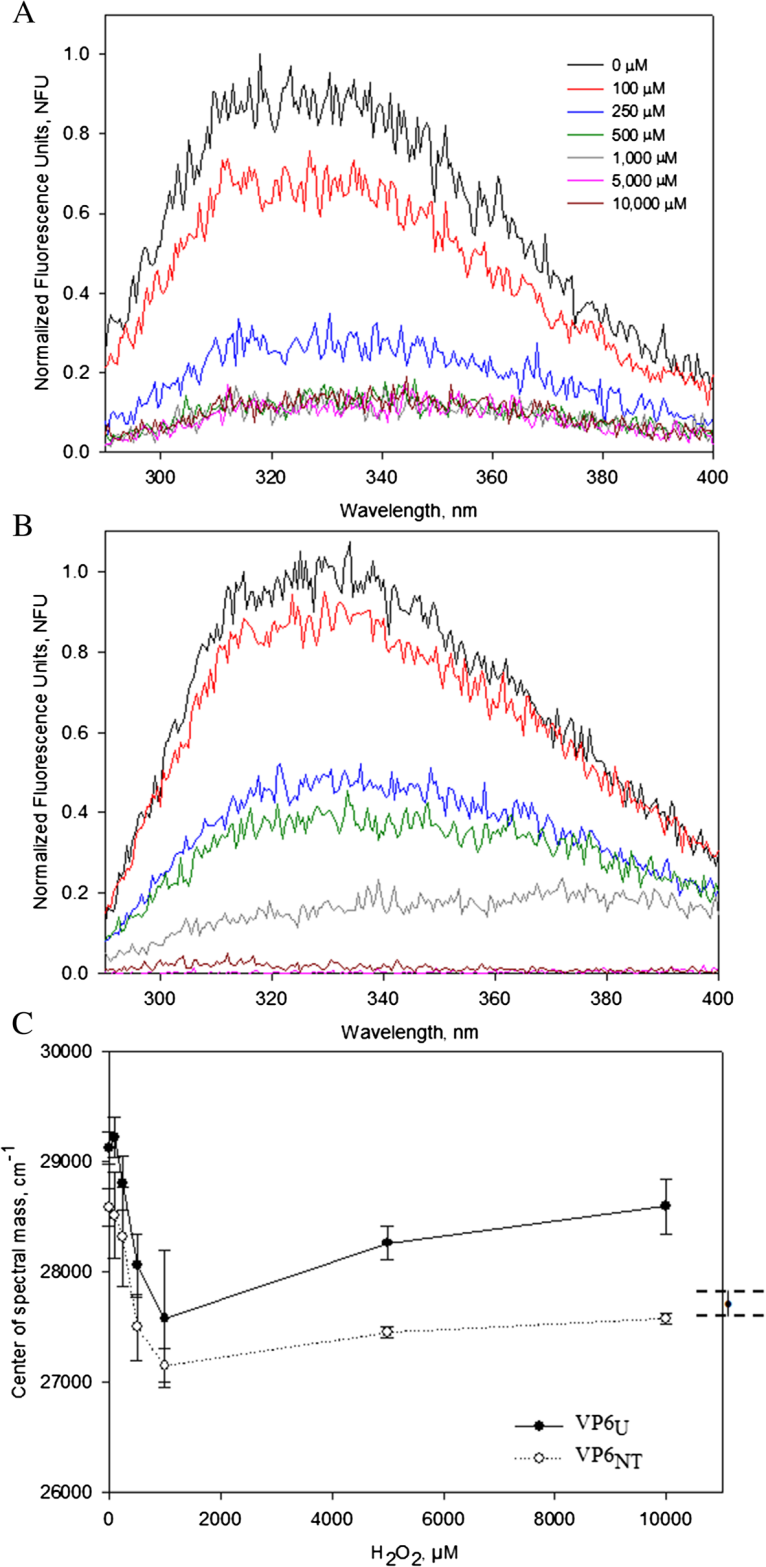
**Emission fluorescence scans at λ**_**ex **_**295 nm of VP6**_**NT **_**and VP6**_**U **_**(0.4 mg/mL) after MCO with 150 µM of FeCl**_**2 **_**at various H**_**2**_**O**_**2 **_**concentrations for 1 h. A)** Normalized emission spectra of VP6_NT_. **B)** Normalized emission spectra of VP6_U_. **C)** The center of spectral mass (CSM) of oxidized VP6_NT_ and VP6_U_ was calculated using Equation (3) and plotted against the H_2_O_2_ concentration used. On the right, the CSM of denatured VP6 (treated for 2 hours with 6 M of guanidine chloride) is shown for comparison. Experiments were performed in triplicate, except for the CSM of denatured VP6, which was analyzed in duplicate. Error bars represent the standard deviation or difference among them.

Fluorescence emission spectra (290 nm to 600 nm) of VP6_U_ and VP6_NT_ subjected to MCO were recorded for Tyr/Trp (λ_ex_ 280 nm, data not shown) and Trp (λ_ex_ 295 nm, Figure 7A and B). Fluorescence of both VP6_U_ and VP6_NT_ decreased as the concentration of H_2_O_2_ increased. Fluorescence decreased the most at H_2_O_2_ concentrations above 0.1 mM, where it diminished at least 50% in all cases. Fluorescence of VP6_NT_ had a more pronounced decrease than fluorescence of VP6_U_, disappearing at H_2_O_2_ concentrations above 250 µM. The center of fluorescence spectral mass (CSM) was calculated for each spectra (Figure 7C). For comparison, the CSM of VP6 treated for 1 h with 6 M guanidine chloride, an unfolded control, is shown at the right of the figure (CSM = 27,642 ± 136 cm^-1^). For both VP6 forms, the CSM decreased as the H_2_O_2_ concentration increased up to 1 mM, and reached wavelengths comparable to that of the unfolded control. At higher H_2_O_2_ concentrations, CSM of VP6_U_ consistently increased.

### Efficiency assembly of VP6_NT_ decreased after oxidation

**Figure 8:**
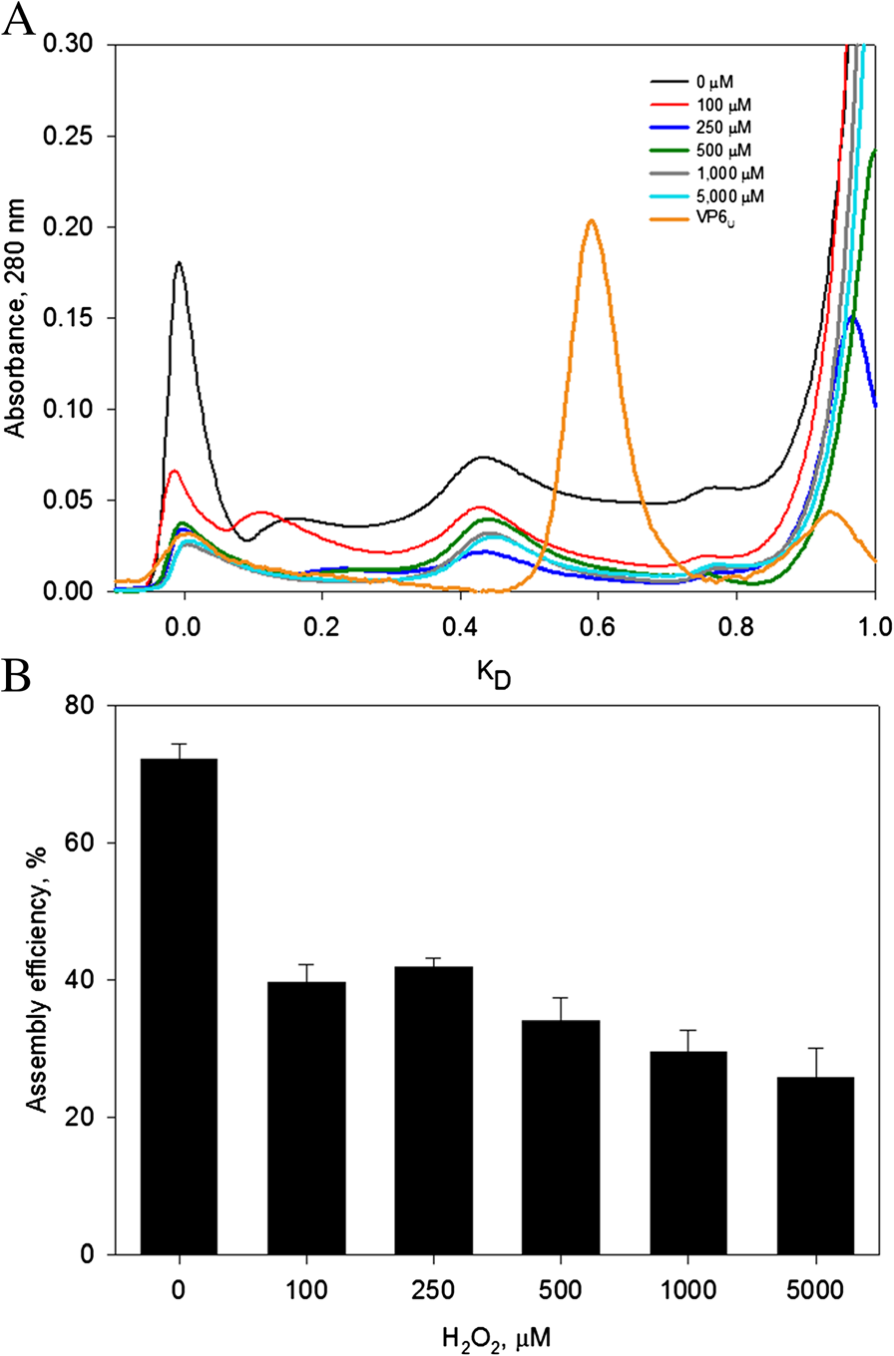
**Assembly efficiency of VP6 exposed to MCO for 1 h (150 µM of FeCl**_**2**_**).** 1 mg of VP6_U_ at 0.4 mg/mL was subjected to assembly conditions. **A)** SEC of samples of VP6 after incubation at the assembly conditions. For comparison, a chromatogram of VP6_U_ is also shown. **B)** Assembly efficiencies calculated from chromatograms in A. Assembly efficiencies were calculated as the area under the curve of peak 1 of each chromatogram divided by the total area below the curve at K_D_ lower or equal to 0.8. Experiments were performed in duplicate, difference between duplicates is represented by error bars.

**Figure 9:**
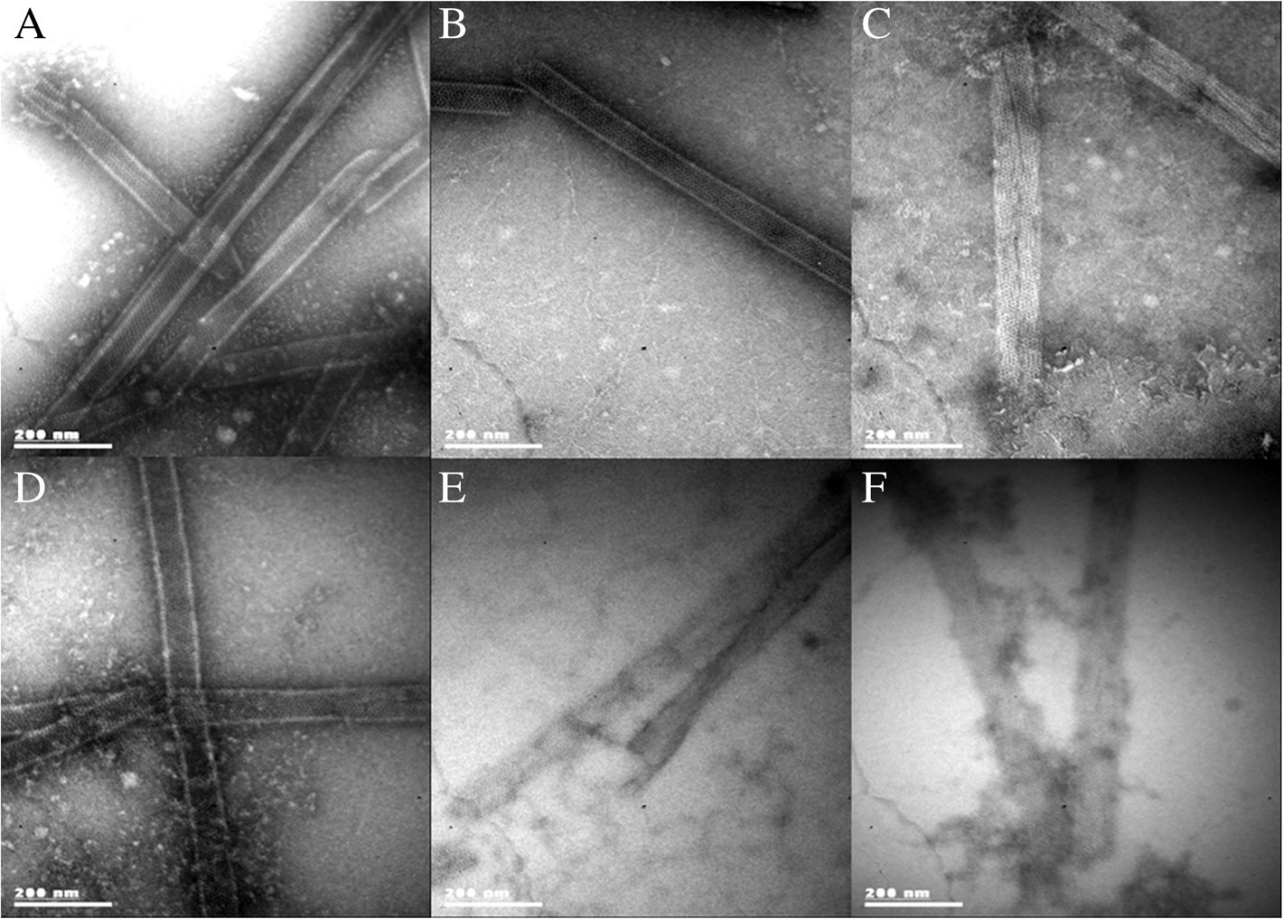
**TEM micrographs of VP6**_**U **_**subjected to assembly conditions after exposure for 1 h to MCO. A)** Nanotubes obtained after assembly of untreated VP6_U_. Other panels, MCO with **B)** 100 µM **C)** 250 µM. **D)** 500 µM. **E)** 1,000 µM. **F)** 5,000 µM of H_2_O_2_. Samples were stained with 2% uranyl acetate and observed at a magnification of 85,000X.

Oxidized VP6_U_ at various H_2_O_2_ concentrations was subjected to *in vitro* assembly conditions to determine if oxidation impedes its assembly into VP6_NT_. Samples incubated for 6 h were analyzed by SEC to determine the relative concentration of assembled VP6, which elutes with the void volume of the SEC column (K_D_ = 0) (Figure 8A). A sample of VP6_U_ not treated for assembly is shown for comparison. VP6_U_ eluted at a K_D_ of 0.6. The peak corresponding to VP6_U_ disappeared in all samples treated for assembly, and peaks appeared at lower K_D_. The abundance of the peak corresponding to VP6_NT_ decreased as H_2_O_2_ concentration increased. Assembly efficiencies were determined by SEC and calculated by dividing the area of the peak at 0 K_D_ (corresponding to VP6_NT_) by the total peak area below 0.8 K_D_, to exclude the salt peak (Figure 8A). VP6_U_ before being subjected to the assembly reactions is shown for comparison. Untreated VP6 assembled into VP6_NT_ with a 72% efficiency. Nanotubes with the expected characteristic were observed by TEM (Figure 9A). The assembly efficiency of oxidized VP6_U_ decreased as H_2_O_2_ concentration increased (Figure 8B), and the quality of the assembled VP6_NT_ decreased (Figure 9B to F). It was increasingly difficult to find assembled nanotubes as the H_2_O_2_ concentration increased, and those found had various defects, such as fractures, incomplete NT and association with aggregates (Figure 9B to F). Most likely the assembly efficiency was overestimated, as aggregation of VP6_U_ upon oxidation was observed.

## Discussion

The importance of oxidation in the integrity of macromolecules has been widely recognized [7]. However, only one previous article has reported the effect of oxidation in a VLP, focusing mostly on the immunogenicity of a vaccine [24]. To study the effect of oxidation on protein assemblies, rotavirus VP6 in two forms, unassembled and assembled into nanotubes, was subjected to oxidation. BSA, a widely studied protein, was treated in parallel for comparison. Protein degradation, carbonylation, size, appearance assessed by TEM, spectrophotometric characterization, and assembly efficiencies were evaluated to determine the effect of oxidation on protein assemblies.

The experiments performed here showed that BSA is less resistant to oxidation than VP6. BSA was degraded by MCO at lower H_2_O_2_ concentrations and shorter times than VP6. Degradation of BSA by oxidation has been previously observed in SDS-PAGE gels similar to those shown here [15]. Protein degradation is a consequence of peptide bond cleavage. It has been proposed that α-amidation and diamide formation are the main mechanisms of peptide bond rupture under oxidative conditions [34-36].

Protein carbonylation is the most destructive irreversible modification caused by oxidation. It is also an excellent biomarker of oxidative stress because of its early formation and stability [37,38]. BSA was less resistant to carbonylation than VP6. Carbonyl contents found here were similar to those previously reported for BSA [15,36]. The higher susceptibility of BSA to damage by oxidation is probably the result of its higher content of lysine, arginine, proline and threonine (RKPT), 149 residues/mol, in comparison with the 82 residues/mol in VP6. The side chains of RPKT amino acids are the most important precursors of carbonylated compounds [34,39]. The constants obtained from data fitting to Equation 1 provide information about the carbonyl content at H_2_O_2_ saturation and the susceptibility of the protein to carbonylation. At 150 µM of Fe^+2^ and 10,000 µM of H_2_O_2_, the carbonyl content in BSA was close to saturation. Also, the value of *a* for BSA was the lowest, in accordance with the experimental observations that BSA was less resistant to oxidation than VP6 in either of its assembled forms. In addition to its higher number of RKPT residues, BSA has 35 cysteines, forming 17 disulfide bonds, while VP6 has only 8 cysteines and no disulfide bonds. Cysteines are also highly susceptible to oxidation, forming a wide variety of compounds [40].

The fluorescence emission of unassembled and assembled VP6 showed that the microenvironment of Tyr and Trp residues are different in both assemblies. The quantum yield of VP6_U_ was twice that of VP6_NT_, possibly because Trp residues are exposed to the solvent only in VP6_U_ (as visualized from the crystal structure reported previously [26]). While for VP6_U_, the Tyr and Trp residues exposed to the solvent are 18 and 8, respectively, for VP6_NT_ only 3 Tyr are exposed [25].

The experiments performed here show that the extent of oxidation of assembled VP6 lower than that of VP6_U_, suggesting that number of oxidizable amino acids that are exposed to the solvent is more important for overall protein oxidation than chain reactions that can be triggered by ROS and propagate the damage to the protein assembly. VP6 assembly into nanotubes may result in additional protection to VP6, as the VP6 nanotube lumen is not freely accessible to ions [28]. In the two sets of carbonylation reactions performed, VP6_U_ had the highest maximum carbonyl content, supporting the idea that a higher protein area exposed to the solvent results in more oxidation. However, the value of *a* from Equation 1 was lower for VP6_NT_, indicating that even when fewer amino acids were oxidized, they were more readily accessible to ROS. The higher susceptibility of VP6_NT_ to oxidation was accompanied by a steeper decrease in fluorescence, suggesting that the aromatic amino acids exposed to the surface were the more susceptible to oxidation. The maximum carbonyl content obtained in VP6_U_ was similar to the number of amino acids in VP6. As certain amino acids are more susceptible to carbonylation than others, this suggests that each oxidized amino acid generated more than one carbonyl group.

DLS analysis showed that oxidation provoked the disassembly of VP6_NT_ and the aggregation of VP6_U_, probably caused by carbonylation. Changes in hydrophobicity due to carbonylated amino acid residues induce protein aggregation [38]. Also, accumulation of oxidized dysfunctional proteins with reactive carbonyl groups can lead to inter- and intramolecular cross-links with amino groups [41]. No formation of dityrosines was found, indicating that aggregation had a different cause, possibly hydrophobic interactions, hydrogen bonding or free thiol groups cross-linking [31,32]. Aggregation and nanotube disassembly have important impacts on possible applications of VP6_NT_. Aggregation has been linked with protein immunogenicity [42], and nanotubes are required for the use of VP6 as an efficient vaccine or as a delivery vehicle [2,43]. Interestingly, MCO of HBsAg VLP did not produce aggregation even at higher oxidant concentrations (100 mM of H_2_O_2_ and 100 µM of Fe^+2^) than those evaluated in the present work.

Fluorescence emission spectra drastically changed upon oxidation of both VP6 forms. Fluorescence emission decreased as H_2_O_2_ concentration increased. Similar results were described by Davies and coworkers [17], who observed fluorescence quenching of aromatic amino acids, mainly Trp, after oxidation with •OH and •OH + O_2_^–^ radicals. Oxidative damage to proteins includes oxidation of aromatic rings, resulting in non-fluorescent derivatives [44]. CSM also decreased as H_2_O_2_ increased up to 1 mM of H_2_O_2_, moving towards the CSM of the unfolded VP6 control, most likely caused by a partial denaturation of VP6 [45,46]. The CSM at 295 nm correlates with the microenvironment surrounding Trp residues, which can be in a relaxed structure (hydrophilic, lower CSM values) or in a compact structure (hydrophobic, higher CSM values) [45,47]. The CSM of VP6_NT_ remained lower than that of VP6_U_ at all conditions tested, indicating that aromatic amino acids remained in a more hydrophilic environment in VP6_NT_ than in VP6_U_. CSM of both VP6 forms increased at H_2_O_2_ concentrations above 1 mM, probably as a result of aggregation.

Oxidation decreased the assembly capacity of VP6_U_ by about half, even at only 100 µM of H_2_O_2_. All the modifications provoked by oxidation described above can result in a lower assembly efficiency of VP6. Either conformational changes or modification of the amino acids in intratrimer or intertrimer regions can impede assembly. For example, Erk and coworkers [48] replaced the His 153 of VP6 with Ser, resulting in a protein that can assemble into trimers but not into nanotubes. Moreover, aggregation provoked by oxidation can also reduce the assembly efficiency into nanotubes. Interestingly, oxidation resulted in nanotubes that had several defects, indicating that even when oxidized VP6 could assemble, the resulting assemblies were less stable. The results obtained highlight the importance of impeding oxidation during the production, purification and storage of protein assemblies and their subunits.

## Conclusions

In this work, the role of oxidation on multimeric protein assembly was described for the first time. Differential effects of the susceptibility of oxidation of a protein assembled or unassembled were found. The results presented here show that oxidation can cause important changes to assembled and unassembled VP6, affecting the protein functionality. *In vitro* assembly of VP6_U_ to form VP6_NT_ decreased with oxidation, evidencing that ROS have to be minimized during the production process when VP6_NT_ are needed. *In vitro* studies of protein oxidation are useful tools for development of new bioprocess to reduce the impact of oxidation on therapeutic proteins produced in heterologous systems. The results of this work show that oxidation must to be avoided in all production stages, including upstream, downstream, handling and storage.

## Methods

### VP6 nanotube production, purification and characterization

VP6 nanotubes were produced using the insect cell-baculovirus expression vector system (IC-BVS) as described before [29]. Briefly, High Five® insect cells (Life Technologies, Carlsbad, CA, USA) were grown in 1 L shake flasks with 200 mL of Sf900II medium (Life Technologies, Carlsbad, CA, USA). Cells were infected at 1 × 10^6^ cell/mL with a recombinant baculovirus (AcMNPV) that contains a rotavirus VP6 gene (strain SA11), at a multiplicity of infection (MOI) of 1 plaque forming unit (pfu)/cell. Infected cultures were harvested at 96 hours post infection (hpi) and centrifuged at 10,000 × g for 10 minutes. Clarified supernatants were concentrated by ultrafiltration using a nitrocellulose membrane with a 30 kDa cut-off (Merck, Billerica, MA, USA). Purification of VP6 assembled as nanotubes (VP6_NT_) was performed as described previously [29]. Total protein content was determined using the Bradford assay (Bio-rad Laboratories, Hercules, CA, USA) and sample purity was calculated from densitometry of reducing denaturing SDS-PAGE gels. VP6 identity was confirmed by Western blot using a polyclonal rabbit serum against NCDV rotavirus (1:4000 dilution in PBS-T), an anti-rabbit IgG-HRP antibody (Santa Cruz Biotechnology, Santa Cruz, CA, USA) (1:4,000 in PBS-T) and developed with carbazole. A prestained molecular weight marker, Dual Color, was used for Western blot experiments (Bio-rad Laboratories, Hercules, CA, USA).

The presence of VP6 assemblies, such as VP6_NT_, was detected by size exclusion HPLC (SEC) [29,33] in a Waters chromatographic system (Waters Corp., MA, USA) with an UV diode array detector at 205, 260 and 280 nm and a fluorescence detector set at λ_ex_ 280 nm and λ_em_ 350 nm (for aromatic amino acid fluorescence). Protein separation was performed using an Ultrahydrogel 500 size exclusion column (Waters Corp., MA, USA) with an isocratic flow of 0.9 mL/min of Tris buffer (10 mM, pH 8.0). The size exclusion column was calibrated with purified protein standards of lysozyme (hydrodynamic radius, R_H_ = 1.8 nm), trypsinogen (R_H_ = 2.2 nm), green fluorescence protein (R_H_ = 2.4 nm), ovoalbumin (R_H_ = 2.8 nm), bovine serum albumin (R_H_ = 3.5 nm), mouse immunoglobulin G (5.3 nm), and 30 nm standard fluorospheres (λ_ex_ = 505 nm, λ_em_ = 515 nm, Life Technologies, Carlsbad, CA, USA). The column void volume (V_0_) was determined with 100 nm standard fluorospheres and the total column volume (V_t_) was determined with sodium azide. The partition coefficient (K_D_) of each protein was calculated using the following equation:

(2) $$K_{D}=\frac{V_{e}‐V_{o}}{V_{t}‐V_{o}}$$

where V_e_ is the elution volume of the protein peak.

The hydrodynamic size of VP6_NT_ was determined by dynamic light scattering (DLS) in a Zetasizer Nano (Malvern Inst. Ltd, Worcestershire, UK) at 173° backscatter using a normal resolution mode. Sizes are reported as the diameter of the equivalent sphere of the particles analyzed. Samples for transmission electron microscopy (TEM) were placed over 200 mesh copper grids coated with Formvar-carbon (Structure Probe Inc., West Chester, PA, USA) and stained with 2% uranyl acetate (Structure Probe Inc., West Chester, PA, USA) for 1 min and visualized in a Zeiss EM 900 transmission electron microscope (Carl Zeiss Microscopy GmbH, Jena, Germany) operated at 80 KV.

### Disassembly and assembly of VP6

VP6_NT_ were disassembled by adding 300 mM of Ca^+2^ and incubating for 6 h at 27°C with constant agitation in a Thermomixer Comfort (Eppendorf, Hauppauge, NY, USA) [33]. For reassembly, VP6 samples at 0.4 mg/mL were mixed with 10 volumes of 100 mM sodium bicarbonate (Sigma Aldrich, St. Louis, USA) at pH 8.0 to precipitate calcium and centrifuged at 5,000 rpm for 10 min. Supernatants were collected and concentrated through a 30 kDa cut-off membrane in an Amicon ultrafiltration device (Merck Millipore Corp, Billerica, MA, USA).

### Protein oxidation

VP6_NT_ and VP6_U_ were oxidized with two oxidants, H_2_O_2_ (Fermont, Monterrey, Mexico) or •OH (obtained through the Fenton reaction, Fe^+2^/H_2_O_2_), using various H_2_O_2_ concentrations (50, 100, 250, 500, 1,000, 2,500, 5,000 and 10,000 µM). For the Fenton reaction, a constant concentration of 150 µM of Fe^+2^ (as FeCl_2_, Sigma Aldrich, St. Louis, USA) was maintained, unless otherwise noted. In some experiments, Fe^+2^/H_2_O_2_ in equimolar concentrations were used. Oxidation with •OH is referred as metal-catalyzed oxidation (MCO) throughout the text. Samples were incubated at 27°C for 1 and 6 h. Oxidized VP6_NT_ and VP6_U_ were analyzed by SDS-PAGE (denaturing and non-denaturing conditions), SEC, DLS and TEM. 2.5 µg of protein were used for each MCO condition in SDS-PAGE experiments.

### Detection and quantification of protein carbonylation

Protein carbonylation was detected by immunoblotting using the Oxyblot™ kit (Merck Millipore Corp, Billerica, MA, USA), following manufacturer instructions: 1 µg of protein was incubated with 2,4-dinitrophenylhydrazine (DNPH) (Sigma Aldrich, St. Louis, USA) for 25 minutes, followed by addition of 0.5% β-mercaptoethanol (Sigma Aldrich, St. Louis, USA). The reaction products were resolved by SDS-PAGE and transferred to a nitrocellulose membrane. The membrane was incubated with a rabbit anti-DNP antibody (1:300 dilution in PBS-T), and a goat anti-rabbit-HRP antibody (1:2000 dilution in PBS-T) (Santa Cruz Biotechnology, Santa Cruz, CA, USA). A molecular weight marker, containing oxidized proteins, was loaded into all gels as a positive control. Densitometric analyses were performed using the ImageJ Software (NIH, USA).

Total carbonyl content was quantified using the method described by Guedes and coworkers [15], with some modifications: 40 to 80 µg of VP6 were derivatized with 160 µL DNPH (10 mM) for 1 h at room temperature in the dark, then samples were washed three times with TCA (20%) and centrifuged at 12,000 x g for 20 min. Supernatants were discarded and pellets were washed three times with 160 µL of ethanol/ethyl acetate solution (1:1, v/v) to remove DNPH excess. Finally, the pellet was dissolved in 100 µL of 6 M guanidine hydrochloride and incubated at 37°C for 10 min. Absorbance was measured at 370 nm in a Nanodrop 1000 spectrophotometer (Thermo Fisher Scientific, USA). The carbonyl content was calculated using an absorption coefficient of 22,000 M^-1^ cm^-1^[36].

### Dityrosine formation and intrinsic fluorescence of aromatic amino acids

Dityrosine formation was detected using fluorescence emission at 320 to 500 nm, at an excitation wavelength of 315 nm, with a slit width of 2.5 nm and a scan speed of 50 nm/min. Temperature was controlled at 27°C. Data were acquired with the FLWinlab software (Perkin Elmer Instruments, MA; USA).

Fluorescence scans were performed with a Luminiscence spectrometer LS55 (Perkin Elmer Instruments, MA, USA) at excitation wavelengths of 280 or 295 nm using a slit width of 2.5 nm. Emission spectra were recorded from 280 to 600 nm using a slit width of 2.5 nm and a scan speed of 50 nm/min. Temperature was controlled at 27°C. Data were acquired with the FLWinlab software (Perkin Elmer Instruments, MA; USA) and the center of fluorescence spectral mass (CSM) was calculated using the following equation [45]:

(3) $$\mathrm{CSM}=\frac{\sum_{280\;\mathrm{nm}}^{500\;\mathrm{nm}}v_{i}*\mathrm{RFU}}{\sum_{280\;\mathrm{nm}}^{500\;\mathrm{nm}}\mathrm{RFU}}$$

where *v*_*i*_ represents the wavenumber (cm^-1^) and RFU the relative fluorescence units.

## Abbreviations

AAPH: 2,2′-azobis(2-amidinopropane) dihydrochloride; BSA: Bovine serum albumin; c: Carbonyl; CSM: Center of fluorescence spectral mass; DLS: Dynamic light scattering; HBsAg: Hepatitis B surface antigen; KD: Partition coefficient; MCO: Metal catalyzed oxidation; p: Protein; RFU: Relative fluorescence units; ROS: Reactive oxygen species; SEC: Size exclusion chromatography; TEM: Transmission electron microscopy; V0: Column void volume; Vt: Total column volume; VP6NT: VP6 nanotubes; VP6U: Unassembled VP6.

## Competing interests

The authors declare that they have no competing interests.

## Authors’ contributions

RMCA performed the study design and the experimental work, participated in the data analysis and manuscript writing. WARL and BV participated in the study design, data analysis and manuscript writing. OTR participated in the study design, data analysis and critically revised the manuscript. LAP conceived and coordinated the study, participated in the study design, data analysis and manuscript writing. All authors read and approved the final manuscript.

## References

[B1] Palomares LA Ramírez OT Challenges for the production of virus-like particles in insect cells: the case of rotavirus-like particles Biochem Eng J 2009 45 158 167 10.1016/j.bej.2009.02.006

[B2] Pastor AR Rodríguez-Limas WA Contreras MA Esquivel E Esquivel-Guadarrama F Ramírez OT Palomares LA The assembly conformation of rotavirus VP6 determines its protective efficacy against rotavirus challenge in mice Vaccinein press 10.1016/j.vaccine.2014.02.018 24583002

[B3] Plascencia Villa G Saniger JM Ascencio JA Palomares LA Ramírez OT Use of recombinant rotavirus VP6 nanotubes as a multifunctional template for the synthesis of nanobiomaterials functionalized with metals Biotechnol Bioeng 2009 104 871 881 10.1002/bit.22497 19655393

[B4] Rodríguez-Limas WA Sekar K Tyo KE Virus-like particles: the future of microbial factories and cell-free systems as platforms for vaccine development Curr Opin Biotechnol 2013 24 1089 1093 10.1016/j.copbio.2013.02.008 23481378 PMC7127385

[B5] Hawe A Wiggenhorn M van de Weert M Garbe JH Mahler H Jiskoot W Forced degradation of therapeutic proteins J Pharm Sci 2012 101 895 913 10.1002/jps.22812 22083792

[B6] Li S Schöneich C Borchardt RT Chemical instability of protein pharmaceuticals: mechanisms of oxidation and strategies for stabilization Biotechnol Bioeng 1995 48 490 500 10.1002/bit.260480511 18623513

[B7] Torosantucci R Schöneich C Jiskoot W Oxidation of therapeutic proteins and peptides: structural and biological consequences Pharm Res 2013 1 13 http://dx.doi.org/10.1007/s11095-013-1199-9 24065593 10.1007/s11095-013-1199-9

[B8] Meucci E Mordente A Martorana G Metal-catalyzed oxidation of human serum albumin: conformational and functional changes. Implications in protein aging J Biol Chem 1991 266 4692 4699 2002018

[B9] Dean R Fu S Stocker R Davies M Biochemistry and pathology of radical-mediated protein oxidation Biochem J 1997 324 1 18 9164834 10.1042/bj3240001 PMC1218394

[B10] Barelli S Canellini G Thadikkaran L Crettaz D Quadroni M Rossier JS Tissot JD Lion N Oxidation of proteins: basic principles and perspectives for blood proteomics Proteom Clin Appl 2008 2 142 157 10.1002/prca.200780009 21136821

[B11] Goetz ME Luch A Reactive species: a cell damaging rout assisting to chemical carcinogens Cancer Lett 2008 266 73 83 10.1016/j.canlet.2008.02.035 18367325

[B12] Baron CP Refsgaard HH Skibsted LH Andersen ML Oxidation of bovine serum albumin initiated by the Fenton reaction-effect of EDTA, tert-butylhydroperoxide and tetrahydrofuran Free Radic Res 2006 40 409 417 10.1080/10715760600565752 16517506

[B13] Liu D Ren D Huang H Dankberg J Rosenfeld R Cocco MJ Li L Brems DN Remmele RL Jr Structure and stability changes of human IgG1 Fc as a consequence of methionine oxidation Biochemistry 2008 47 5088 5100 10.1021/bi702238b 18407665

[B14] Hu D Qin Z Xue B Fink AL Uversky VN Effect of methionine oxidation on the structural properties, conformational stability, and aggregation of immunoglobulin light chain LEN Biochemistry 2008 47 8665 8677 10.1021/bi800806d 18652490 PMC2676884

[B15] Guedes S Vitorino R Domingues R Amado F Domingues P Oxidation of bovine serum albumin: identification of oxidation products and structural modifications Rapid Commun Mass Sp 2009 23 2307 2315 10.1002/rcm.4149 19575405

[B16] Mulinacci F Capelle MAH Gurny R Drake AF Arvinte T Stability of human growth hormone: influence of methionine oxidation on thermal folding J Pharm Sci 2011 100 451 463 10.1002/jps.22293 21249719

[B17] Davies K Delsignore M Lin S Protein damage and degradation by oxygen radicals. II. Modification of amino acids J Biol Chem 1987 262 9902 9907 3036876

[B18] Hawkins CL Davies MJ Generation and propagation of radical reactions on proteins BBA-Bioenerg 2001 1504 196 219 10.1016/S0005-2728(00)00252-8 11245785

[B19] Sano D Pintó RM Omura T Bosch A Detection of oxidative damages on viral capsid protein for evaluating structural integrity and infectivity of human norovirus Environ Sci Technol 2009 44 808 812 20000802 10.1021/es9018964

[B20] Meunier S Strable E Finn M Crosslinking of and coupling to viral capsid proteins by tyrosine oxidation Chem Biol 2004 11 319 326 10.1016/j.chembiol.2004.02.019 15123261

[B21] Rule Wigginton K Menin L Montoya JP Kohn T Oxidation of virus proteins during UV_254_ and singlet oxygen mediated inactivation Environ Sci Technol 2010 44 5437 5443 10.1021/es100435a 20553020

[B22] Bounty S Rodriguez R Linden KG Inactivation of Adenovirus Using Low-Dose UV/H_2_O_2_ Advanced Oxidation Water Res 2012 46 6273 6278 10.1016/j.watres.2012.08.036 23040991

[B23] Ogata N Inactivation of influenza virus haemagglutinin by chlorine dioxide: oxidation of the conserved tryptophan 153 residue in the receptor-binding site J Gen Virol 2012 93 2558 2563 10.1099/vir.0.044263-0 22933663

[B24] Tleugabulova D Falcón V Pentón E Sewer M Fleitas Y Aggregation of recombinant hepatitis B surface antigen induced in vitro by oxidative stress J Chromatogr B 1999 736 153 166 10.1016/S0378-4347(99)00453-3 10676995

[B25] Lepault J Petitpas I Erk I Navaza J Bigot D Dona M Vachette P Cohen J Rey FA Structural polymorphism of the major capsid protein of rotavirus EMBO J 2001 20 1498 1507 10.1093/emboj/20.7.1498 11285214 PMC145494

[B26] Mathieu M Petitpas I Navaza J Lepault J Kohli E Pothier P Prasad BV Cohen J Rey FA Atomic structure of the major capsid protein of rotavirus: implications for the architecture of the virion EMBO J 2001 20 1485 1497 10.1093/emboj/20.7.1485 11285213 PMC145492

[B27] Blazevic V Lappalainen S Nurminen K Huhti L Vesikari T Norovirus VLPs and rotavirus VP6 protein as combined vaccine for childhood gastroenteritis Vaccine 2011 29 8126 8133 10.1016/j.vaccine.2011.08.026 21854823

[B28] Carreño-Fuentes L Ascencio JA Medina A Aguila S Palomares LA Ramírez OT Strategies for specifically directing metal functionalization of protein nanotubes: constructing protein coated silver nanowires Nanotechnology 2013 24 235602 10.1088/0957-4484/24/23/235602 23676195

[B29] Plascencia-Villa G Mena JA Castro-Acosta RM Fabián JC Ramírez OT Palomares LA Strategies for the purification and characterization of protein scaffolds for the production of hybrid nanobiomaterials J Chromatogr B 2011 879 1105 1111 10.1016/j.jchromb.2011.03.027 21474396

[B30] Giulivi C Davies KJ Dityrosine: a marker for oxidatively modified proteins and selective proteolysis Methods Enzymol 1994 233 363 371 8015471 10.1016/s0076-6879(94)33042-5

[B31] Mahler HC Friess W Grauschopf U Kiese S Protein aggregation: pathways, induction factors and analysis J Pharm Sci 2009 98 2909 2934 10.1002/jps.21566 18823031

[B32] Auer S Dobson CM Vendruscolo M Characterization of the nucleation barriers for protein aggregation and amyloid formation HFSP J 2007 1 137 146 10.2976/1.2760023 19404419 PMC2639838

[B33] Mena JA Ramírez OT Palomares LA Quantification of rotavirus-like particles by gel permeation chromatography J Chromatogr B 2005 824 267 276 10.1016/j.jchromb.2005.07.034 16095985

[B34] Stadtman E Levine R Free radical-mediated oxidation of free amino acids and amino acid residues in proteins Amino Acids 2003 25 207 218 10.1007/s00726-003-0011-2 14661084

[B35] Garrison WM Reaction mechanisms in the radiolysis of peptides, polypeptides, and proteins Chem Rev 1987 87 381 398 10.1021/cr00078a006

[B36] Headlam HA Davies MJ Markers of protein oxidation: different oxidants give rise to variable yields of bound and released carbonyl products Free Radic Bio Med 2004 36 1175 1184 10.1016/j.freeradbiomed.2004.02.017 15082071

[B37] Fedorova M Bollineni RC Hoffmann R Protein carbonylation as a major hallmark of oxidative damage: update of analytical strategies Mass Spectrom Rev 2014 33 79 97 10.1002/mas.21381 23832618

[B38] Dalle-Donne I Rossi R Giustarini D Milzani A Colombo R Protein carbonyl groups as biomarkers of oxidative stress Clin Chim Acta 2003 329 23 38 10.1016/S0009-8981(03)00003-2 12589963

[B39] Rao R Møller IM Pattern of occurrence and occupancy of carbonylation sites in proteins Proteomics 2011 11 4166 4173 10.1002/pmic.201100223 21919202

[B40] Jeong J Jung Y Na S Jeong J Lee E Kim M-S Choi S Shin D-H Paek E Lee H-Y Novel oxidative modifications in redox-active cysteine residues Mol Cell Proteomics 2011 10 M110.000513 10.1074/mcp.M110.000513 21148632 PMC3047142

[B41] Shigenaga MK Hagen TM Ames BN Oxidative damage and mitochondrial decay in aging Proc Natl Acad Sci U S A 1994 91 10771 10778 10.1073/pnas.91.23.10771 7971961 PMC45108

[B42] Singh SK Impact of product-related factors on immunogenicity of biotherapeutics J Pharm Sci 2011 100 354 387 10.1002/jps.22276 20740683

[B43] Rodríguez M Wood C Sanchez-López R Castro-Acosta RM Ramírez OT Palomares LA Understanding internalization of rotavirus VP6 nanotubes by cells: towards a recombinant vaccine Arch Virol In press 10.1007/s00705-013-1916-z 24232915

[B44] Simat T Steinhart H Oxidation of free tryptophan and tryptophan residues in peptides and proteins J Agr Food Chem 1998 46 490 498 10.1021/jf970818c 10554268

[B45] Mohana-Borges R Silva JL de Prat-Gay G Protein folding in the absence of chemical denaturants. Reversible pressure denaturation of the noncovalent complex formed by the association of two protein fragments J Biol Chem 1999 274 7732 7740 10.1074/jbc.274.12.7732 10075663

[B46] Vivian JT Callis PR Mechanisms of tryptophan fluorescence shifts in proteins Biophys J 2001 80 2093 2109 10.1016/S0006-3495(01)76183-8 11325713 PMC1301402

[B47] Lakowicz JR Principles of fluorescence spectroscopy 2009 Springer

[B48] Erk I Huet J-C Duarte M Duquerroy S Rey F Cohen J Lepault J A zinc ion controls assembly and stability of the major capsid protein of rotavirus J Virol 2003 77 3595 3601 10.1128/JVI.77.6.3595-3601.2003 12610135 PMC149495

